# Invalid Children

**Published:** 1887-08-13

**Authors:** 


### Invalid Children.

The Charity Organisation Society, with the object of bringing to the aid of the poor sick children the intelligent sympathy and helpful services of good women, has issued the following circular letter :—

MEDICAL SUB-COMMITTEE OF THE CHARITY ORGANISATION SOCIETY.

15, Buckingham Street, Adelphi, W.C.

*May*, 1886.

Dear Sir,—It has been proposed that I should undertake as Secretary to the Medical Sub-committee of the Charity Organisation Society, a bit of work which it is very desirable to accomplish, and which will, it is believed, meet with the approval and sympathy of many who are outside the ranks of the regular supporters of the Charity Organisation Society.

Those who have had much to do with the poor of London, and especially those who have been in any way connected with hospitals, are aware that there are large numbers of children seriously crippled by spine and hip-joint disease, and other distressing maladies. Anyone walking through the wards of one of our hospitals will find his heart wrung by the spectacle of scores of patient little sufferers whose young lives are prematurely clouded by an affliction, the gravity of which they are mercifully scarcely able to appreciate. And yet here the spectator sees, perhaps, these lives at their best. Let him think what they must in many cases be when the poor invalids go away from their comfortable cots—where to treat them, and nurse them, and cheer them is the reason of everybody's existence—to squalid and crowded rooms, where a struggle with downright want is being waged, and where the helpless can scarcely fail to be regarded as a burden, and hindrance, and misfortune. Even if such love and care as are possible are given, how much they must be without! Much alone, and to a great extent unoccupied, how weary the hours may be! How much must be lacking in the way of material comfort, and how remote the possibility of the treatment and the alleviations which we should in such a case secure at any sacrifice for our own little ones.

And yet the remedy is obvious. If an effectual system of visitation could be set on foot, so that each poor child should have his or her friend, whose task it would be, in every way that human wit and kindness could devise, to make the little life easier and brighter, how great the good that might be done, how much of pain, and hardship, and sadness, might be removed.

I do not propose in this letter to go in great detail into the various modes in which the visitors we have in view would make themselves useful to the children they undertook to look after. The friendly visit, once a fortnight or so, would of itself be a bright spot in the sick child's existence. The more serious cases would be benefited by having as a visitor one able to convey medical suggestions, and to give the mother hints as to the nursing and management of the patient. In some instances little would be wanted, in others much. A proper bed, a perambulator, an occasional drive, suitable diet for a time, a sojourn in the country or by the sea—a long list of ways of helping would not take long to frame.

Our aim is that there should not be in the poorer homes of London a single crippled or afflicted child without some kind friend attached to him, who would bring something of pleasure into his life, and who, being one of a large band of workers, and able to have recourse to an organisation possessing means and experience, would often be in a position to give valuable advice and, where necessary, material help.

It will be seen that the visitors are not themselves expected to incur any great expense. Where they wish to do so, or to get the means of helping from private friends, their co-operation will be thankfully accepted, but they will be at liberty to apply to me for anything of a costly kind that may be required, and it is hoped that the public, as the plan becomes known, will enable the Society to meet all demands.

We believe that there are hundreds—nay, thousands—of men and women to whom this would be congenial work. It need not demand of them more than they can give. A visitor need not of necessity have more than one case under his charge. He need not, unless he prefers it, belong to the Charity Organisation Society. All we ask is to be furnished with the names and addresses of those who will consent to "father" or "mother" one afflicted child, as near as may be to the locality where they live. We ask you to mention the scheme to all your friends, and kindly to send to me the names of any who, you think, might be willing to undertake the work.

May I, above all, enter your name in our book as one of our visitors for this special purpose? If, as I greatly hope, you will consent to this, please send me a line, addressed—Lieut.-Col. Montefiore, 15, Buckingham Street, Strand, W.C.

A little time may elapse before you hear again, as there will be a good deal to arrange for before the work of visitation is actually commenced; but you may rely upon it that no needless delay will be incurred.—Believe me, yours faithfully,

E. Montefiore, Lieut.-Col.

